# Identification of osteoarthritis-characteristic genes and immunological micro-environment features through bioinformatics and machine learning-based approaches

**DOI:** 10.1186/s12920-023-01672-y

**Published:** 2023-10-07

**Authors:** Zheng Da, Rui Guo, Jianjian Sun, Ai Wang

**Affiliations:** 1 Xingtai People’s Hospital Affiliated to Hebei Medical University, Xingtai City, Hebei Province China; 2 Ningbo Huamei Hospital, University of Chinese Academy of Sciences, Ningbo City, Zhejiang Province China; 3 Zhongshan Hospital Affiliated to Fudan University, Shanghai City, China

**Keywords:** Osteoarthritis, Bioinformatics, Cartilage, Biomarkers, Herc5, Tspan2, HtrA1

## Abstract

**Background:**

Osteoarthritis (OA) is a multifaceted chronic joint disease characterized by complex mechanisms. It has a detrimental impact on the quality of life for individuals in the middle-aged and elderly population while also imposing a significant socioeconomic burden. At present, there remains a lack of comprehensive understanding regarding the pathophysiology of OA. The objective of this study was to examine the genes, functional pathways, and immune infiltration characteristics associated with the development and advancement of OA.

**Methods:**

The Gene Expression Omnibus (GEO) database was utilized to acquire gene expression profiles. The R software was employed to conduct the screening of differentially expressed genes (DEGs) and perform enrichment analysis on these genes. The OA-characteristic genes were identified using the Weighted Gene Co-expression Network Analysis (WGCNA) and the Lasso algorithm. In addition, the infiltration levels of immune cells in cartilage were assessed using single-sample gene set enrichment analysis (ssGSEA). Subsequently, a correlation analysis was conducted to examine the relationship between immune cells and the OA-characteristic genes.

**Results:**

A total of 80 DEGs were identified. As determined by functional enrichment, these DEGs were associated with chondrocyte metabolism, apoptosis, and inflammation. Three OA-characteristic genes were identified using WGCNA and the lasso algorithm, and their expression levels were then validated using the verification set. Finally, the analysis of immune cell infiltration revealed that T cells and B cells were primarily associated with OA. In addition, Tspan2, HtrA1 demonstrated a correlation with some of the infiltrating immune cells.

**Conclusions:**

The findings of an extensive bioinformatics analysis revealed that OA is correlated with a variety of distinct genes, functional pathways, and processes involving immune cell infiltration. The present study has successfully identified characteristic genes and functional pathways that hold potential as biomarkers for guiding drug treatment and facilitating molecular-level research on OA.

**Supplementary Information:**

The online version contains supplementary material available at 10.1186/s12920-023-01672-y.

## Introduction

Osteoarthritis (OA) is a highly prevalent musculoskeletal condition on a global scale, and it stands as the primary contributor to disability [1]. OA predominantly impacts the knee joint, and its occurrence is associated with various factors, such as joint injury, joint dysplasia, advancing age, obesity, and genetic predisposition [2]. The quality of life of middle-aged and older individuals can be adversely affected by chronic pain and functional impairment, which can also impose a substantial economic burden on society [3]. OA impacts various anatomical structures within the periarticular region. The process of irreversible articular cartilage destruction may initiate prior to the manifestation of clinical symptoms and radiographic abnormalities [4]. Despite numerous investigations, the pathophysiology and therapeutic options for OA remain elusive. Therefore, the development of innovative biomarkers capable of detecting the degradation of articular cartilage is imperative for the diagnosis and management of OA.

Utilizing high-throughput gene expression profiling, the Weighted Gene Co-expression Network Analysis (WGCNA) method can be employed to discern gene co-expression networks within diseases [5]. This approach facilitates the identification of gene modules that exhibit significant connectivity as well as, the identification of core genes within these modules [6].

**Fig. 1:**
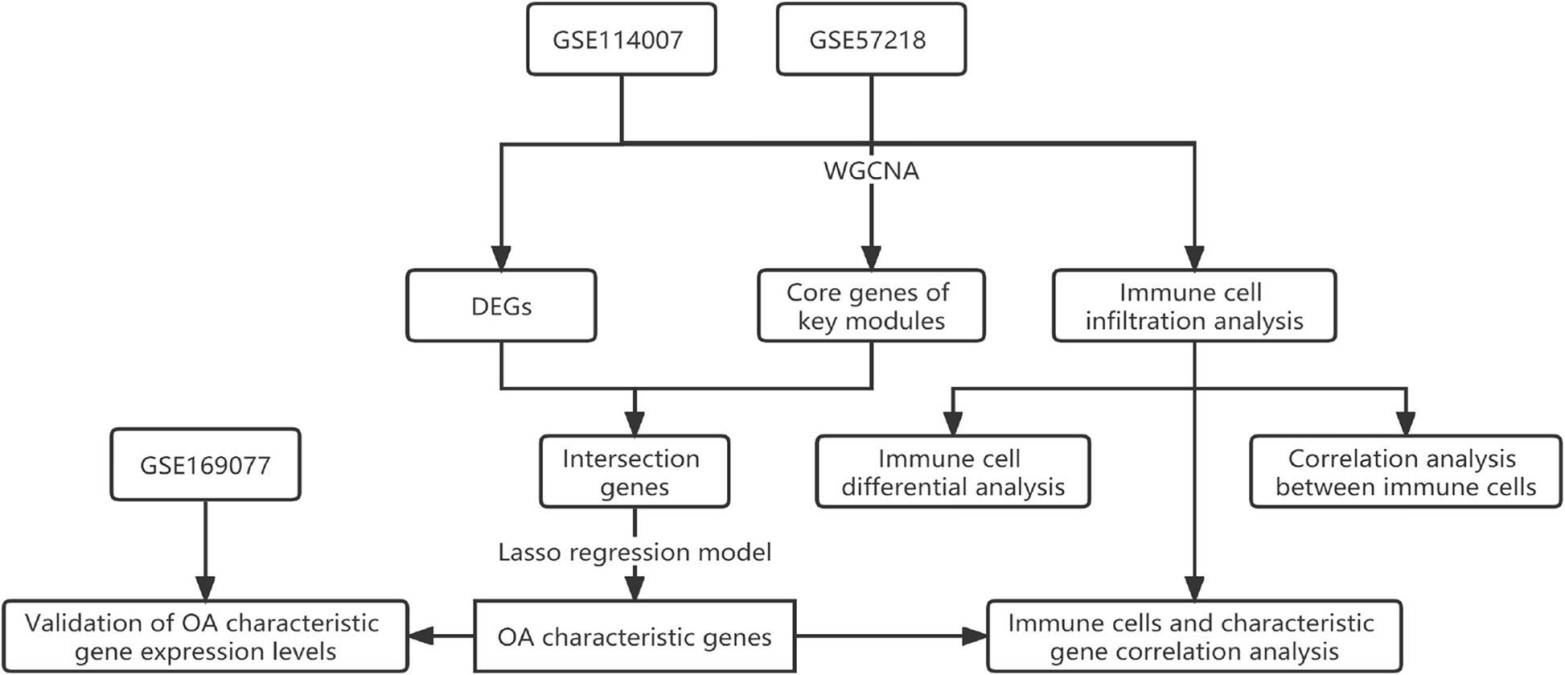
Flowchart of this study

The flowchart for this study is depicted in Fig. 1. First, gene expression profiles of OA cartilage tissues were acquired from the Gene Expression Omnibus (GEO) database. Subsequently, the GSE114007 and GSE57218 datasets were selected and retrieved for further analysis. To identify the differentially expressed genes (DEGs) in healthy and OA-affected cartilage, the combined and corrected datasets were subjected to differential analysis using the limma package in the R software. Subsequently, functional enrichment analyses and the construction of protein-protein interaction networks were performed for the DEGs. The combined dataset was then subjected to WGCNA analysis in order to identify the gene module that is most pertinent to the disease, as well as the core genes within the key module. The core genes derived from WGCNA were compared with DEGs to identify genes that were present in both datasets. Subsequently, the lasso algorithm was employed to acquire the OA-characteristic genes, which were subsequently validated using the GSE169077 dataset. The validation dataset GSE169077 was obtained from the GEO database as well. The present study also encompassed an examination of immune cell infiltration within the samples in order to ascertain the differential infiltration of immune cells in healthy and OA-affected cartilage. Furthermore, the relationship between the OA-characteristic genes and the immune cells was determined via correlation analysis.

This study employed bioinformatics and machine learning techniques to identify the signaling pathways and OA-characteristic genes. In addition, we investigated the relationship between these characteristic genes and immune cells. Furthermore, the identified OA-characteristic genes were validated using additional datasets. Thus, the current study establishes a foundation for understanding the pathogenesis and progression of OA, offering valuable insights for the development of gene-targeted therapies.

## Materials & methods

### Downloading and merging of data sets

The GEO database https://www.ncbi.nlm.nih.gov/GEO/), is a globally recognized public repository that offers free access to high-throughput datasets. The OA cartilage-related gene expression datasets GSE114007, GSE57218, and GSE169077 (Table 1) were obtained through a comprehensive search and subsequent download. It is important to note that GSE114007 and GSE57218 were utilized as training group datasets, while GSE169077 served as the validation group dataset for the present study. The expression matrix of the training group datasets was consolidated, and batch effects were mitigated using the SVA package in R version 4.21 software. As the study was conducted utilizing a publicly available database and did not involve the use of animal or human subjects, it was not necessary to obtain approval from an institutional review board.

**Table 1:** The basic data of each dataset

Database ID	Platform	Author	Year	Tissue	Number of samples	Number of controls
Training set						
GSE57218	GPL6947	Ramos YF	2014	cartilage	33	40
GSE114007	GPL11154	Fisch KM	2018	cartilage	20	18
Validation set						
GSE169077	GPL96	Attur M	2021	cartilage	6	5

### Identification of DEGs

We analyzed gene expression between normal and OA articular cartilage using the limma package in the R programming language based on Bayesian calculations of t-value, f-value, and logarithmic dominance. Correspondingly, we identified the DEGs that between the two groups that met the specified conditions (|logFC| ≥1 and adj. *P*. Val < 0.05). The ggplot2 and pheatmap packages are then utilized to generate a volcanic map and heat map, respectively, in order to visually represent and elucidate the obtained outcomes.

### The functionally enrichment and PPI network construction of DEGs

The Clusterprofiler package in the R programming language was utilized to perform Gene Ontology (GO), Kyoto Encyclopedia of Genes and Genomes (KEGG) [7–9], and Gene Set Enrichment Analysis (GSEA) enrichment analyses. In addition, the PPI network was constructed using the String database (https://cn.string-db.org), with a minimum connectivity score threshold of 0.4.

### Weighted Gene Co-expression Network Analysis (WGCNA)

The WGCNA is a bioinformatics approach utilized for the identification of gene modules associated with diseases. This approach involves the construction of gene co-expression networks and the subsequent identification of significant pathogenic pathways or potential therapeutic targets [5]. The genes that exhibited significant correlations were organized into modules through the application of clustering and dynamic cropping techniques. In order to ascertain gene modules that are linked to OA, we applied a multiplication factor of 60 to the quantity of RNAs within the module set and established a threshold setting of 0.25 as the cut height. The significance of each module was visually represented. The key module, which is most closely linked to OA, was selected for analysis. Core genes within this module were identified based on criteria including a gene significance value > 0.5 and a gene module correlation > 0.80.

### Screening of OA characteristic genes

The identification of overlapping genes was performed by utilizing the Venn package of R version 4.21 software, which involved the integration of DEGs and module-key genes. The LASSO regression model can effectively compress the variable coefficients in a regression model by employing a penalty function. The process of identifying the minimum classification error value (λ) has the potential to lead to the reduction of variable dimensionality. The identification of OA-characteristic genes was performed by screening the acquired overlapping genes, followed by the construction of a lasso regression model using the R programming language.

### Validation of OA characteristic genes in training and testing groups

A comparative analysis was performed to assess the disparity in gene expression in healthy and OA-affected cartilage. The statistical analysis was performed utilizing the limma package in the R programming language. A significance level of 0.05 was adopted for determining statistical significance. The “proc” package of R version 4.21 software was employed to generate receiver operating characteristic (ROC) curves and calculate the area under the curve (AUC) values for healthy and OA-affected samples to investigate the classification effect of the disease-characteristic genes.

### Immune cell infiltration analysis

Single sample gene set enrichment analysis (ssGSEA) was used to evaluate the scores of 28 immune cells to obtain a score file representing the immune cell composition in each individual sample. The immune cell scores of individuals in OA and healthy groups were subjected to statistical analysis using t-tests. The resulting data was then visually represented through the use of violin plots. The association between the disease-characteristic genes and immune cells was examined using the correlation test function in the R software. The results were then visualized using the ggplot package. Subsequently, a correlation analysis was conducted to examine the relationship between each dysregulated immune cell, and the ensuing outcomes were graphically represented.

## Results

### Data preprocessing and differential expression gene screening

**Fig. 2:**
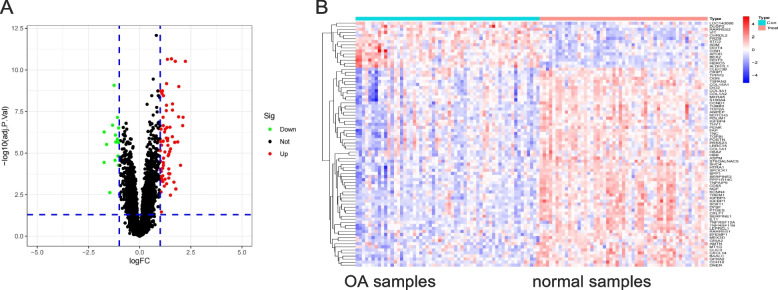
The gene differential expression analysis of GSE57218 and GSE114007 data sets. **A** The DEG volcano map shows up-regulated genes in red and down-regulated genes in green. **B** DEG expression heat map

Following the integration of the datasets GSE57218 and GSE114007, a comprehensive analysis of gene expression profiling was conducted. Based on the principles of differential analysis, a total of 80 DEGs were identified, with 65 genes exhibiting upregulation and 15 genes displaying downregulation (Fig. 2A). Subsequently, a heat map representing the DEGs was generated (Fig. 2B).

### Enrichment analysis of differentially expressed genes

**Fig. 3:**
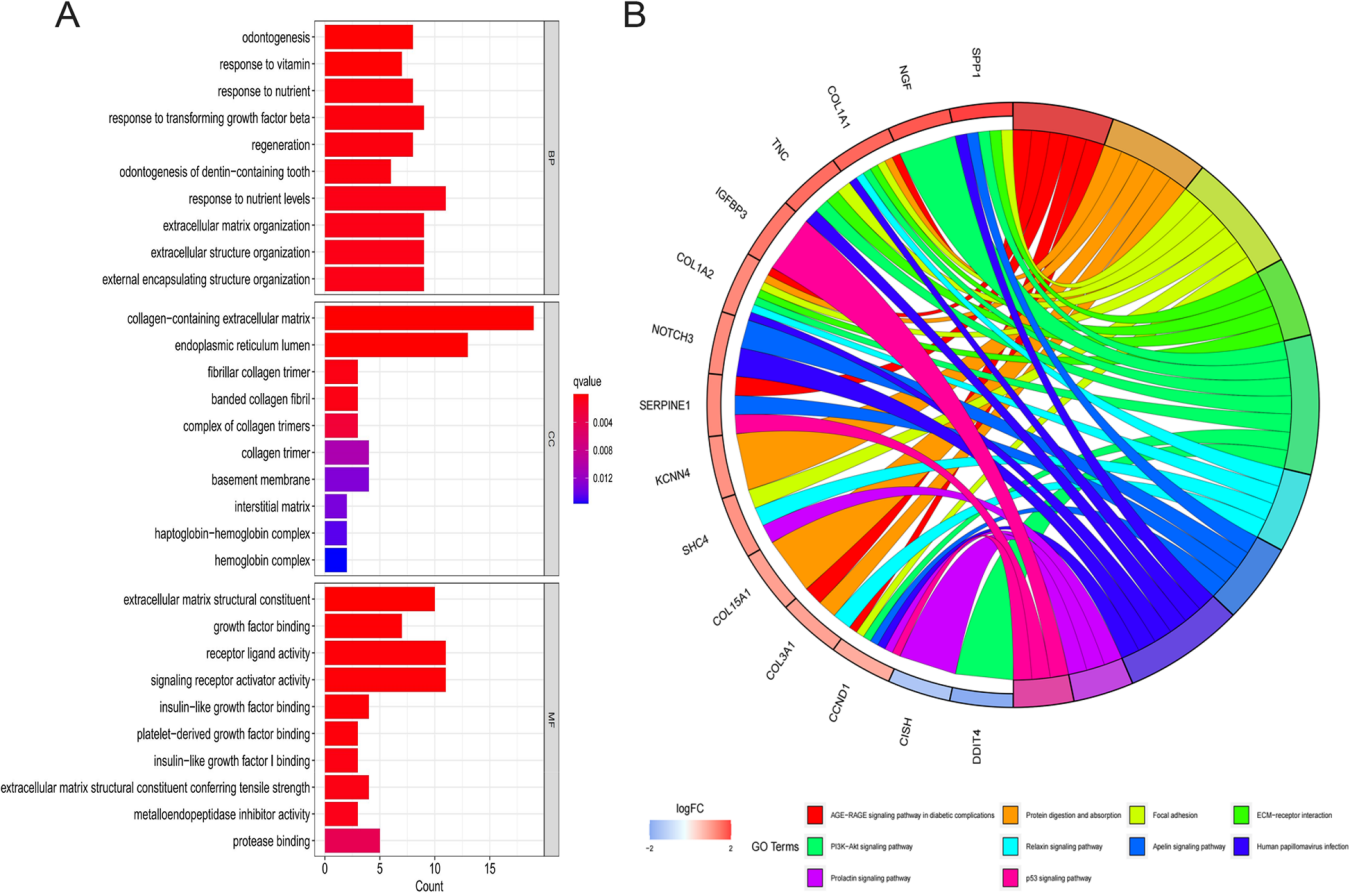
The enrichment analysis of GO and KEGG. **A** Bar chart of the top 10 GO enrichment results. **B** KEGG enrichment circle plot

The DEGs were subjected to analysis using the clusterprofiler package in the R programming language in order to obtain enrichment results for the GO and KEGG pathways. A comprehensive analysis revealed the identification of 93 biological processes (BP), 14 cellular components (CC), 25 molecular functions (MF), and 10 signaling pathways, all of which were found to be statistically significant at a significance level of *P* < 0.05. The outcomes of BP are primarily associated with the organization of the extracellular matrix (ECM), the response to nutrients, the response to nutrient availability, and the organization of extracellular structures, etc. The top 10 enrichment outcomes for MF and CC are shown in Fig. 3A. According to the KEGG results, DEGs are predominantly involved in the ECM-receptor interaction, PI3K-Akt, protein digestion and absorption, focal adhesion, apelin, relaxin, p53, and other signaling pathways (Fig. 3B).

### GSEA analysis and ppi network construction

**Fig. 4:**
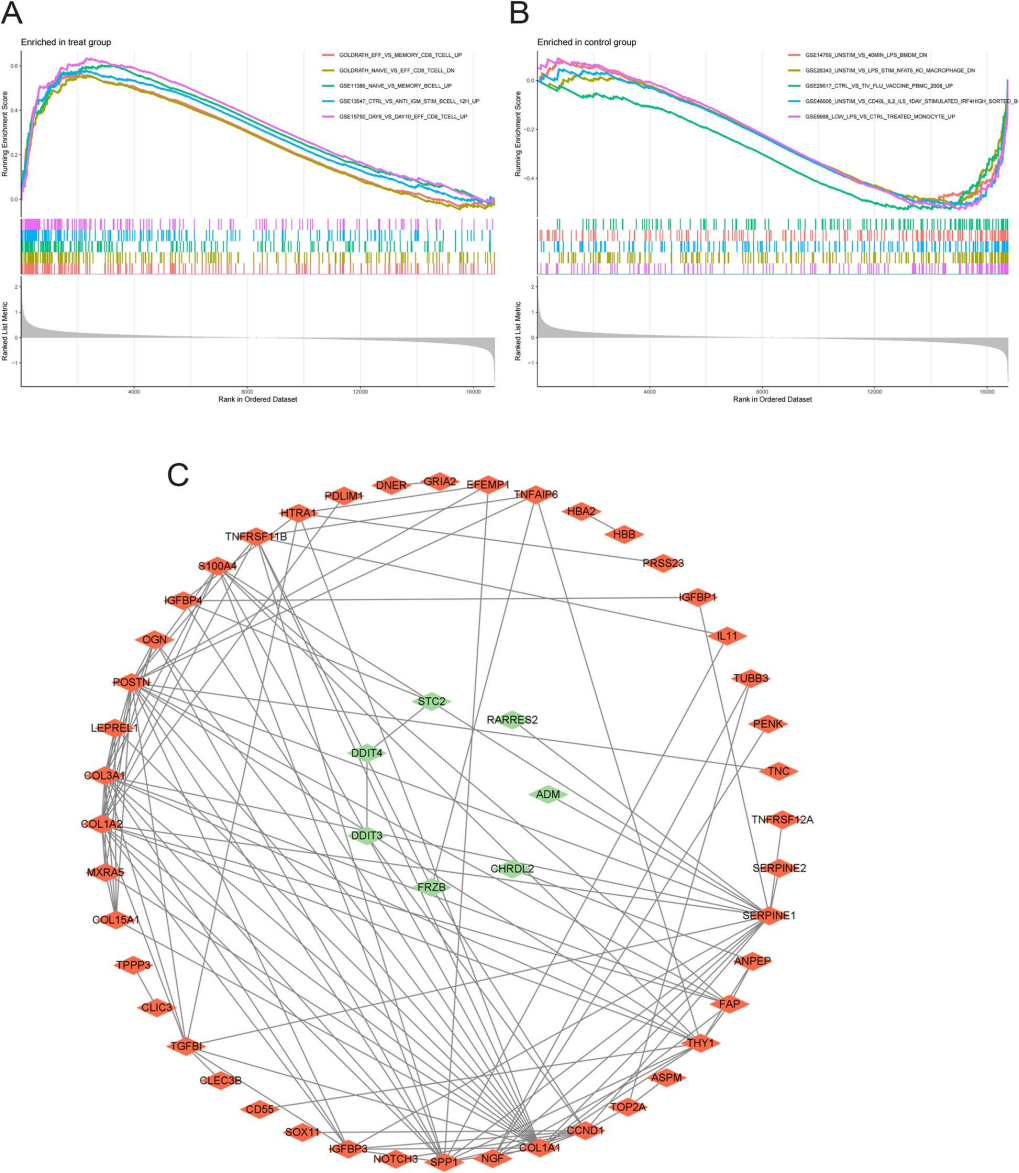
GSEA enrichment analysis and ppi network of DEGs. **A** Gene set active in the OA group. **B** Gene set active in the normal group. **C **Protein-protein interaction (PPI) network, red marks indicate upregulated genes, green marks indicate downregulated genes

The results of the GSEA analysis are presented in Table 2. The gene set of the OA group exhibits significant enrichment in datasets associated with T cells and B cells (Fig. 4A, B). In addition, as shown in Fig. 4C, the PPI network reveals the connections between DEGs and pathways associated with OA.

**Table 2:** Top 5 gene sets that significantly enriched in OA and normal group

Gene set name	NES	*p *value	p.adjust	qvalues
Enriched in OA group.				
GOLDRATH_EFF_VS_MEMORY_CD8_TCELL_UP	2.256176107	1.00E-10	3.25E-08	2.50E-08
GOLDRATH_NAIVE_VS_EFF_CD8_TCELL_DN	2.206479095	1.00E-10	3.25E-08	2.50E-08
GSE11386_NAIVE_VS_MEMORY_BCELL_UP	2.361586552	1.00E-10	3.25E-08	2.50E-08
GSE13547_CTRL_VS_ANTI_IGM_STIM_BCELL_12H_UP	2.265169851	1.00E-10	3.25E-08	2.50E-08
GSE15750_DAY6_VS_DAY10_EFF_CD8_TCELL_UP	2.488187209	1.00E-10	3.25E-08	2.50E-08
Enriched in normal group.				
GSE9988_LOW_LPS_VS_CTRL_TREATED_MONOCYTE_UP	-2.130242333	3.95E-10	1.09E-07	8.41E-08
GSE26343_UNSTIM_VS_LPS_STIM_NFAT5_KO_MACROPHAGE_DN	-2.120165618	5.51E-10	1.41E-07	1.09E-07
GSE46606_UNSTIM_VS_CD40L_IL2_IL5_1DAY_STIMULATED_IRF4HIGH_SORTED_BCELL_DN	-2.098150587	1.44E-09	3.35E-07	2.58E-07
GSE14769_UNSTIM_VS_40MIN_LPS_BMDM_DN	-2.0569626	2.57E-09	5.21E-07	4.01E-07
GSE29617_CTRL_VS_TIV_FLU_VACCINE_PBMC_2008_UP	-2.112167615	3.72E-09	6.71E-07	5.17E-07

### Weighted Gene Co-expression Network Analysis (WGCNA)

**Fig. 5:**
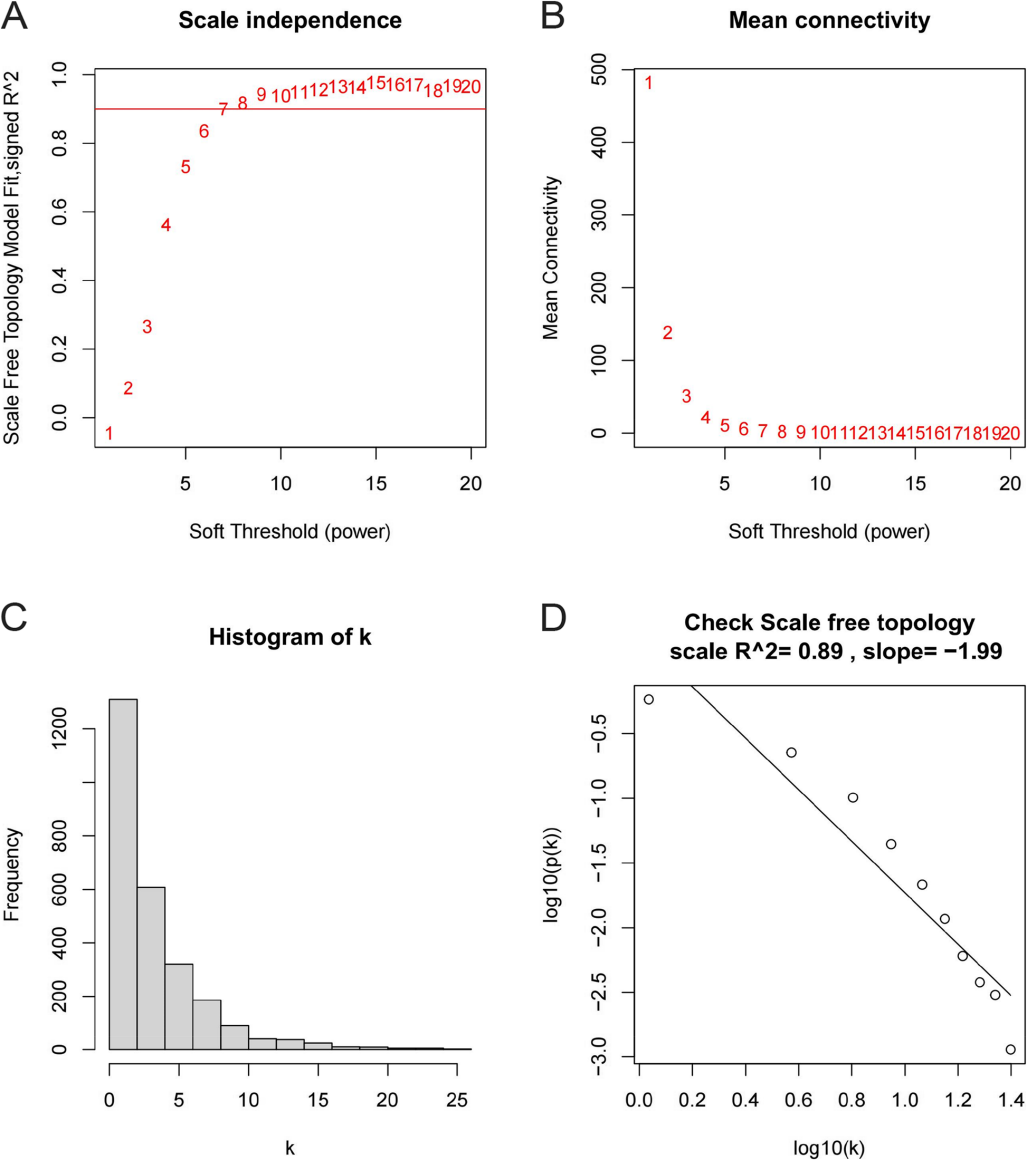
The screening criteria of WGCNA. **A** Relationship between soft threshold and scale-free topological fitting index. **B** Relationship between average network connectivity and scale-free topological fitting index. **C** The distribution of the node connectivity K. **D** Graph of the K-P(K) correlation

The WGCNA was conducted on the merged data set, and a value of β = 7 was selected in order to achieve a suitable fit of the gene distribution to a scale-free network. Sample trees and soft thresholds were constructed based on connectivity degrees, as depicted in Fig. 5A and B, respectively. The vertical axes of the graph depict the scale-free topology fit index R^2 and mean connectivity, while the relationship between these variables and the soft threshold is illustrated. The observed negative correlation between the variables K and P (k) (correlation coefficient = 0.89) suggests that selecting β = 7 satisfies the requirements for constructing a gene scale-free network (Fig. 5 C, D).

**Fig. 6:**
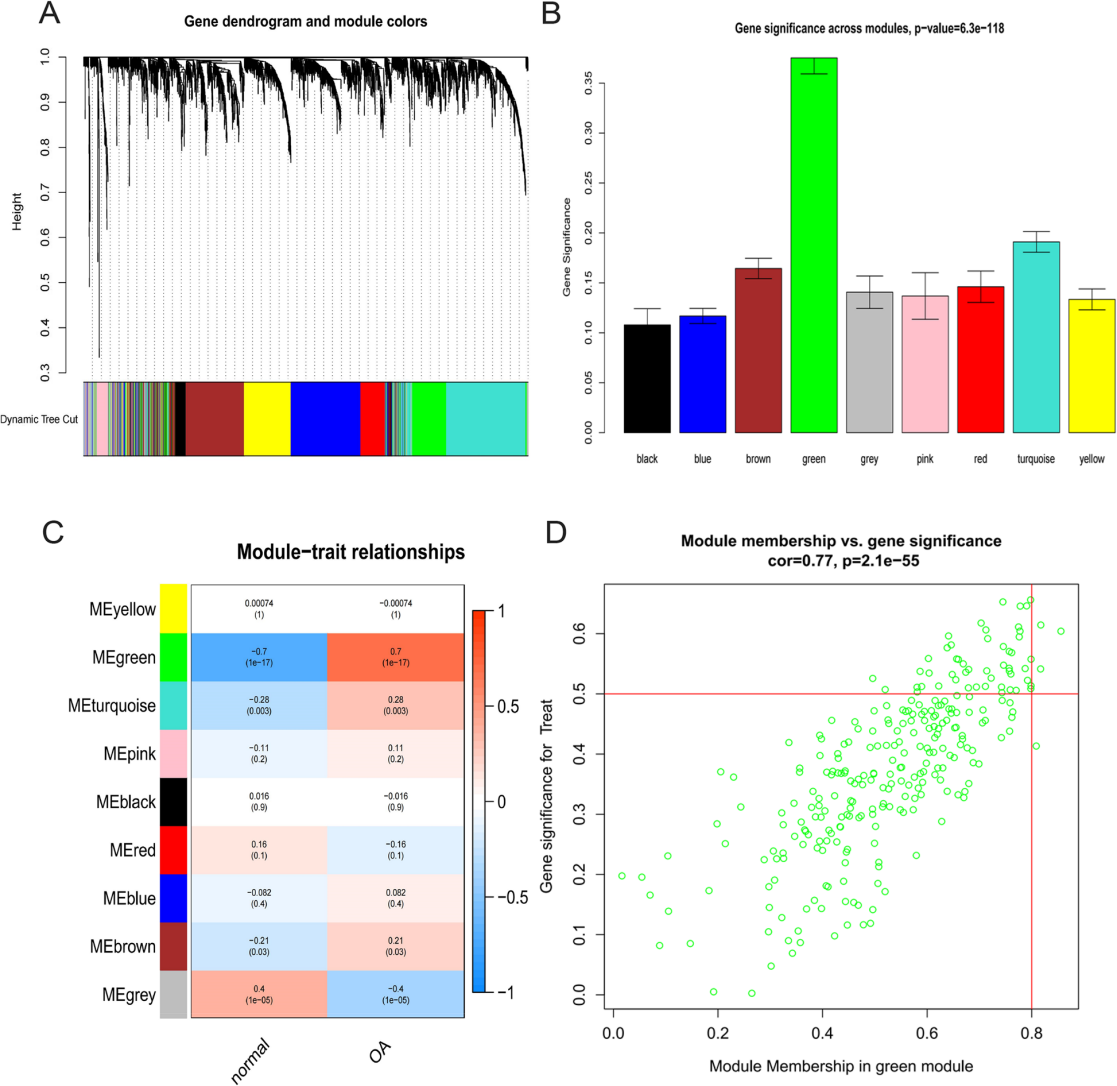
Screening core genes of key modules. **A** Clustering dendrogram of genes. **B** Bar graphs of module gene significance score. **C** Module-trait correlation Heatmap. **D** Scatter plot of green module genes

Nine modules were identified in the gene expression tree and module eigen correlation heatmap following the conversion of the gene expression matrix into an adjacency and topological matrix (Fig. 6A, B). A score was assigned to each module to determine the importance of gene signatures (Fig. 6C). Moreover, the green module, which received the highest score, was identified as the key module. The three central genes of the principal module were examined (Fig. 6D) using the criteria of gene importance > 0.5 and gene module correlation > 0.8.

### Screening of OA characteristic genes

**Fig. 7:**
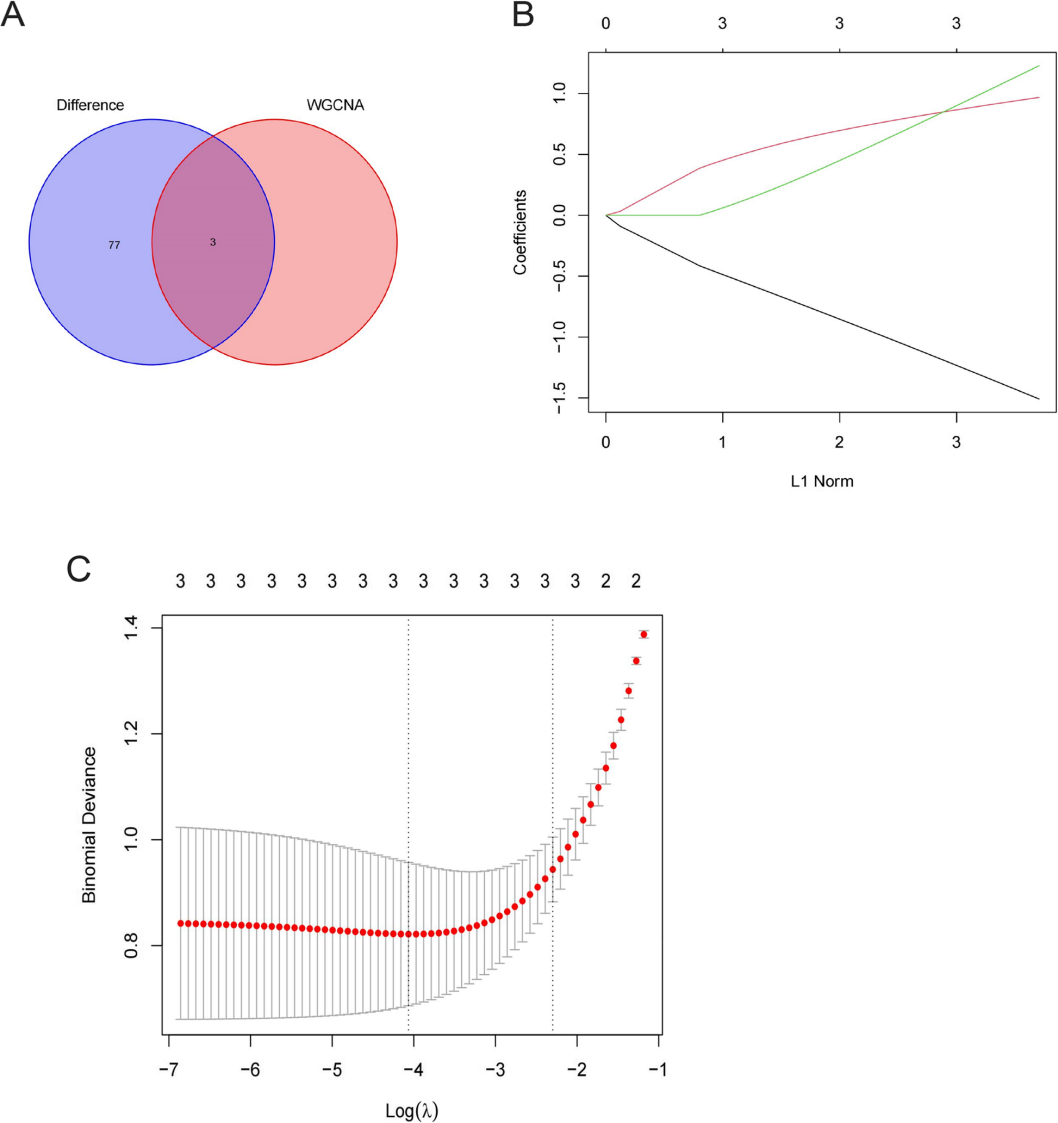
Screening of disease characteristic genes. **A** Obtaining intersecting genes between DEGs and module core genes. **B** LASSO regression coefficients trend .**C** Screening of coordination in LASSO model

Three overlapping genes were identified by intersecting the module core genes with DEGs using the R software (Fig. 7A). Following the application of the lasso regression algorithm, three distinct genes exhibiting substantial discriminatory capabilities between healthy and OA-affected samples were successfully identified (Fig. 7B, C).

### Validation of OA characteristic genes in training and testing groups

**Fig. 8:**
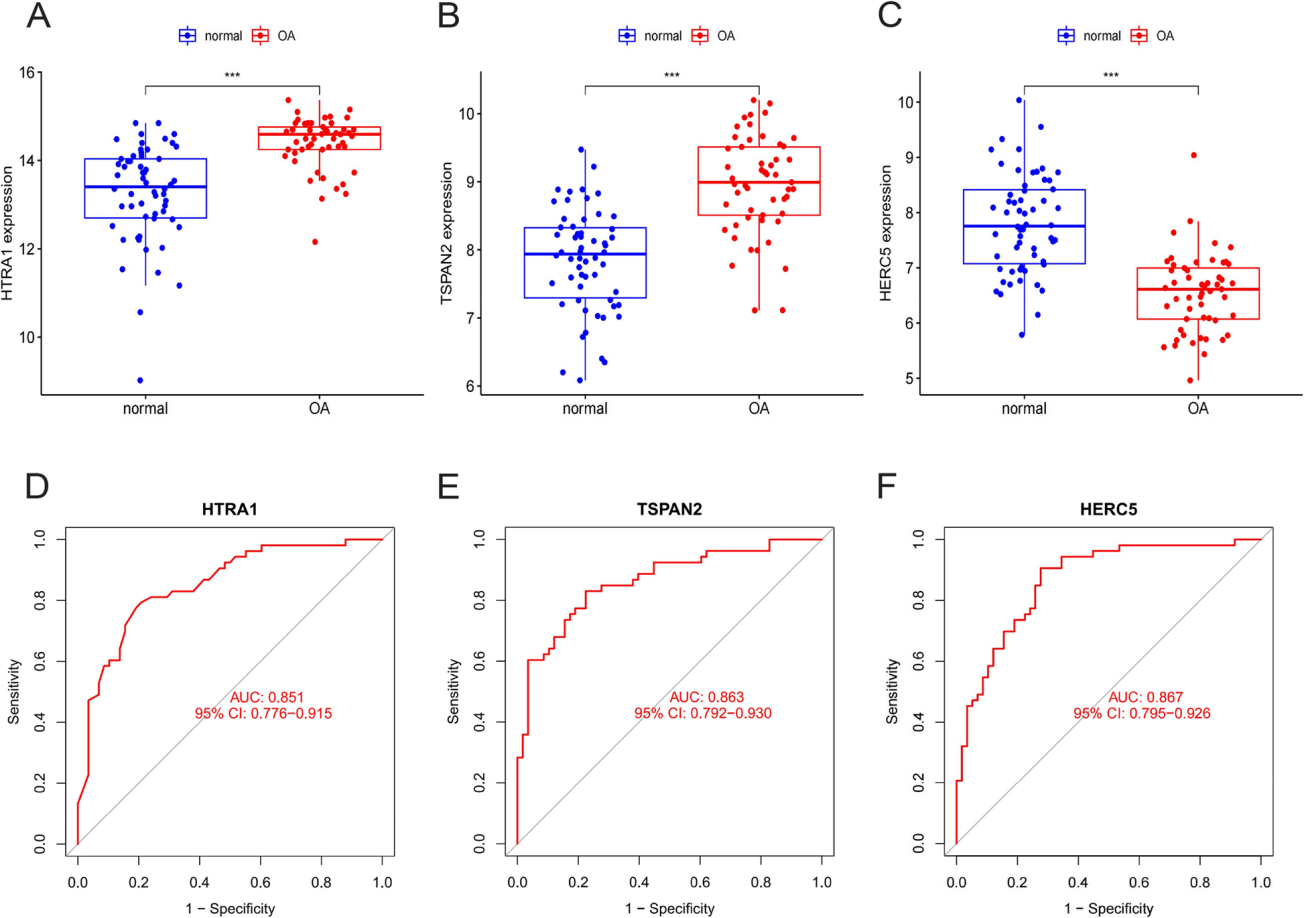
The expression of HERC5, TSPAN2 and HTRA1 and their significance for diagnosis in the training group: **A** The expression of HTRA1 in OA samples was clearly increased. **B** The expression of TSPAN2 in OA samples was clearly increased. **C** Samples from OA displayed distinct downregulation of HERC5. **D**, **E**, **F** HTRA1, TSPAN2 and HERC5 ROC assays in OA

Based on the findings obtained by our research team, it was observed that the OA samples exhibited elevated expression levels of HtrA1 and Tspan2 compared to the healthy samples within the training group (Fig. 8A, B). Conversely, a notable down-regulation of Herc5 was observed in the OA-affected samples (Fig. 8C). In order to enhance our comprehension of the diagnostic efficacy of HtrA1, Tspan2, and Herc5, we conducted ROC analyses on these three biomarkers. The AUC values for the three OA characteristics examined in this study are > 0.85, indicating a robust ability to differentiate OA samples from normal samples (Fig. 8D, E, and F).

**Fig. 9:**
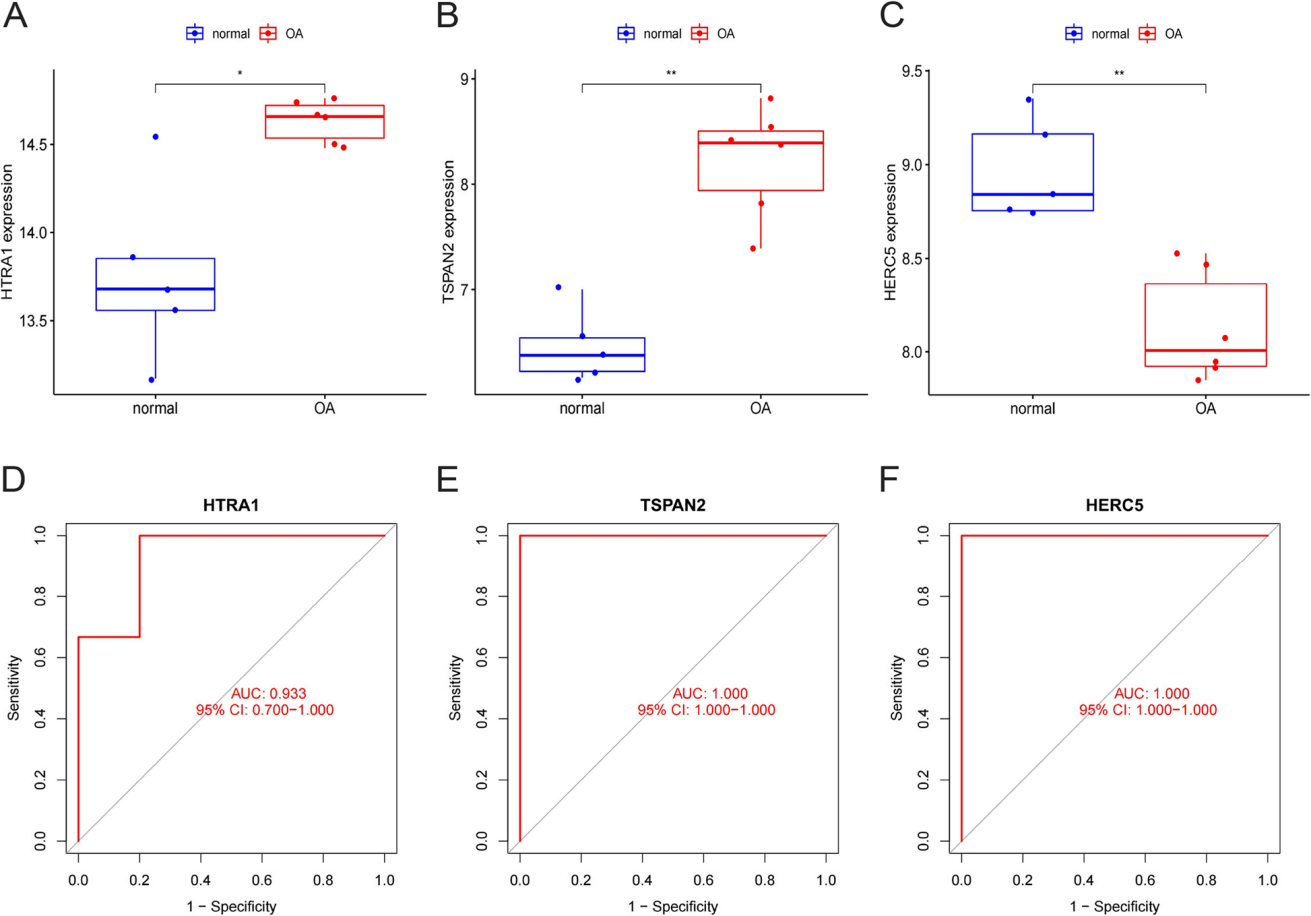
HTRA1, TSPAN2 and HERC5 expression and diagnosis significance in the Validation cohort: **A** Samples from OA patients showed a distinct increase in HTRA1 expression. **B** Samples from OA patients showed a distinct increase in TSPAN2 expression. **C** A significant decrease in HERC5 expression was observed in OA samples. **D**, **E**, **F** The ROC analysis of HTRA1, TSPAN2 and HERC5 in OA

The GSE169077 dataset was selected for the purpose of validating the expression of specific genes associated with OA, viz. Herc5, Tspan2, and HtrA1 in both healthy and OA-affected cartilage. This validation was conducted through the analysis of differential gene expression (Fig. 9A, B, and C) and through ROC analysis (Fig. 9D, E, and F). The research team observed a notable increase in the expression levels of HtrA1 and Tspan2, while the expression levels of Herc5 were observed to be lower in cartilage associated with OA. The findings exhibited congruity with the outcomes observed in the training cohort. Based on our research findings, it can be concluded that HtrA1, Tspan2, and Herc5 possess significant potential as biomarkers for the diagnosis of OA.

### Infiltrated immune cells were correlated with Tspan2, HtrA1

**Fig. 10:**
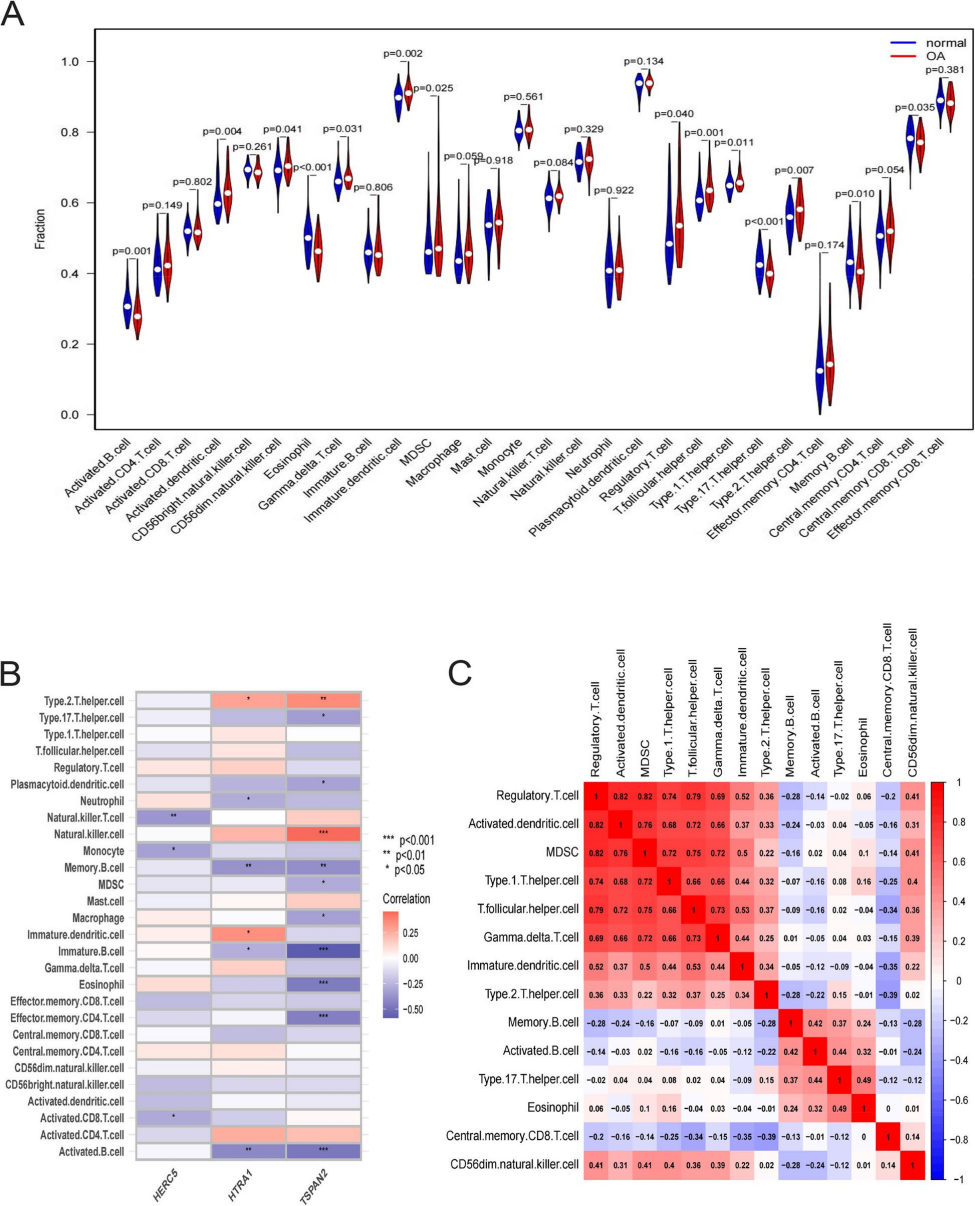
Analysis of immune cell infiltration. **A** Immunocyte architecture differs between healthy and OA cartilage. **B** Heatmap of correlation analysis between immune cells and OA Characteristic Genes. **C** Heatmap of immune cells correlation analysis

The characteristics of immune cells were also investigated by employing the cryptosort method. The study yielded data on immune cell infiltration, specifically the quantification of 28 distinct immune cell types within cartilage samples. Figure 10 (A) displays the outcomes of the comparative analysis conducted between the OA and control groups. Dysregulated levels were observed in various cell types within the OA samples, including activated dendritic cells, immature dendritic cells, Th1 cells, Th2 cells, Th17 cells, T follicular helper cells, Gamma delta T cells, regulatory T cells, central memory CD8 T cells, activated B cells, memory B cells, eosinophils, myeloid-derived suppressor cells (MDSC), and CD56dim natural killer cells. Figure 10 (B, C) illustrate the correlation between immune cells and OA-characteristic genes (Herc5, Tspan2, and HtrA1). Additionally, it depicts the interrelationship among these dysregulated immune cells. The relationship between Tspan2 and dysregulated immune cells involves the activation of B cells, memory B cells, γδT cells, and Th2 cells. Similarly, dysregulated immune cells associated with HtrA1 involve activated B cells, memory B cells, eosinophils, Th2 cells, and Th17 cells. The findings of our study, therefore, indicate that Tspan2 and HtrA1 exhibit interactions with various immune cells, thereby contributing to the progression of OA.

## Discussion

OA is a chronic degenerative joint disorder that manifests as joint pain, inflammation, rigidity, and progressive loss of mobility, severely affecting the quality of life for the middle-aged and elderly populace. In addition to synovitis, the primary characteristics of this condition encompass the degeneration and destruction of articular chondrocytes [1–4]. One of the primary roles of cartilage is to effectively absorb mechanical forces by means of its ECM [10]. The ECM is a highly dynamic, complex molecular network primarily consisting of hyaluronic acid, collagen fibers, and proteoglycans [11, 12]. These constituents play a crucial role in preserving the normal structure and function of cartilage [11, 12]. The joint undergoes a series of molecular pathway dysfunctions during the progression of OA, leading to an imbalance in proteolysis. This imbalance ultimately leads to the degradation of cartilage’s structural integrity and biomechanical properties [13]. Furthermore, these alterations take place prior to the degradation of cartilage [14].

Herein, enrichment analyses using GO and KEGG were performed on a set of 80 DEGs derived from samples of individuals with OA and healthy controls. Based on the results of the GO enrichment analysis, it is evident that DEGs exhibit a prominent presence in various biological processes, including extracellular matrix organization, extracellular structure organization, response to transforming growth factor beta, external encapsulating structure organization, response to nutrient levels, and other related processes. Furthermore, the findings of this study indicate that the DEGs were predominantly enriched in two cellular compartments: the extracellular matrix, which contains collagen, and the lumen of the endoplasmic reticulum. The MF indicated that the DEGs were implicated in various biological processes, including extracellular matrix structural constituents, growth factor binding, receptor-ligand activity, signaling receptor activator activity, and protease binding. In addition, the findings from the GO enrichment analysis indicated that the OA cartilage exhibited more pronounced alterations in the extracellular matrix and a greater degree of involvement in diverse biological processes compared to healthy cartilage. Moreover, the KEGG enrichment analysis indicates that the focal adhesion and PI3K-Akt signaling pathways exhibit the highest count of enriched genes. Adhesion is one of the major ECM receptor-interacting signaling pathways in which integrins play a crucial role. When ECM binds to integrins, focal adhesion kinase (FAK) in cells is phosphorylated. Following its activation, FAK participates in a diverse range of cellular processes, encompassing cell migration, cell differentiation, matrix remodeling, growth factor signaling, and cell survival [15, 16]. Thus, the integrin-FAK interaction was confirmed to be essential in preserving chondrocyte homeostasis [17]. The PI3K-Akt signaling pathway is a very complex pathway comprising over 150 proteins, which plays a crucial role in various cellular processes such as inflammation, metabolism, cell cycle regulation, cell survival, and programmed cell death [18]. These processes are essential for cellular homeostasis [19–21]. The PI3K-Akt signaling pathway exerts a suppressive influence on ECM catabolism. The activation of PI3K-Akt by TGF-b leads to a decrease in the expression of MMP13 [22]. A separate study also demonstrated that IGF-1 activates PI3K and extracellular signal-regulated kinases (ERK), resulting in an increased expression of COL2A1 and the inhibition of MMP13 [23]. Moreover, previous studies have confirmed the significance of the PI3K-Akt signaling pathway in the regulation of chondrocyte survival and apoptosis. Under various pathological conditions, the activation of signals has been observed to have the ability to hinder chondrocyte apoptosis, thereby restricting the advancement of OA [24]. Additional pathways implicated in the pathogenesis of OA have been elucidated through pertinent investigations. These pathways encompass protein digestion and absorption, ECM-receptor interaction, Apelin, and the p53 signaling pathway. The aforementioned pathways indicate a strong correlation between the metabolism, inflammatory response, and apoptosis process of chondrocytes and OA [25–28]. It is imperative to acknowledge that certain pathways, such as the relaxin pathway, hold significant potential for research. Several studies have indicated that relaxin receptors are present in the ligament, cartilage, and synovium of the trapeziometacarpal joint (TM), thereby exerting an influence on the stability of the joint. The potential reason for this phenomenon may be attributed to its involvement in the regulation of collagen, as suggested by previous research [29]. Furthermore, the GSEA revealed a notable enrichment of OA cells in datasets associated with T-cells and B-cells, surpassing the enrichment observed in normal chondrocytes. This suggests a significant impact of inflammation on the progression of OA. Moreover, the findings of extensive enrichment analyses suggest that various pathways, including inflammation, metabolism, apoptosis, and others, may play a role in the processes associated with OA. Furthermore, molecules that have the ability to participate in multiple pathways concurrently may hold promise as potential targets for therapeutic interventions.

Three genes, viz. HtrA1, Tspan2, and Herc5, were identified as having the strongest association with OA through the utilization of two machine learning algorithms. Herc5, a protein with multiple domains and a molecular weight of 114 kDa, belongs to the HEC E3 ubiquitin ligase and RCC1 superfamily. It serves as the primary E3 ligase responsible for the modification of ISG15 [30, 31]. According to reports, there is evidence suggesting that ISG15 has the ability to interact with p53. Additionally, the down-regulation of Herc5 expression has been observed to indirectly facilitate the degradation of p53, resulting in an increase in apoptosis. This process has been well-studied in cancer [32]. Recent research has suggested that Herc5 may also be influenced by IL-1β and TNF-α through distinct signal transduction pathways [33]. Furthermore, the downregulation of Herc5 triggers the activation of the IL-17 A pathway, a crucial component in the inflammatory response, leading to an upregulation of CXCL13, CXCL15, and CXCL16 expression [34]. According to our investigation, it was observed that the expression of Herc5 was reduced in chondrocytes affected by OA. This particular modification could potentially be linked to the occurrence of apoptosis and the inflammatory reaction in individuals with OA. While the immunological importance of Herc5 has been extensively validated, its precise involvement in the pathogenesis of OA remains unclear and necessitates additional research. Tetraspanin 2 (Tspan2), which belongs to the tetraspanin superfamily [35], has been identified on the plasma membrane as well as intracellular organelle membranes in a wide range of cell and tissue types. This regulatory mechanism plays a crucial role in the regulation of cancer, the immune system, and infectious diseases [36, 37]. Tspan2 is responsible for the regulation of protein transportation, specifically its partner proteins such as CXCL-12 and CXCR-4. Additionally, Tspan2 is linked to the expression of IL-13 and IL-10 [35]. Tspan2 has the ability to interact with PIK3-R3 and contribute to the PIK3 signaling pathway [36]. Furthermore, Tspan2, being a cytokine with proapoptotic properties, has the ability to induce cell apoptosis through the activation of the JNK, Wnt, and Akt signaling pathways [38]. The results of our analysis indicate that there was an increase in tspan2 expression in OA cartilage when compared to samples from healthy individuals. Therefore, it is postulated that Tspan2 potentially participates in various chemokine, metabolic, and apoptotic pathways in the progression of OA; however, additional research is required. HtrA1 serves as the primary protease in human OA chondrocytes, effectively initiating catabolic pathways that lead to the deterioration of cartilage integrity [39, 40]. This degradation is facilitated through the cleavage of the fibronectin fragment within the ECM [16]. Furthermore, aside from its intrinsic metabolic function, the indirect activation of HtrA1 and subsequent protein cleavage can be facilitated by the activation of other proteases or the inhibition of HtrA1 protease inhibitors [39]. Moreover, previous studies have demonstrated that the expression of HtrA1 is increased during the initial stages of cartilage degeneration or in cases of minor damage. This finding implies that the expression of HtrA1 takes place during the initial phases of OA [41]. The findings of our study, thus, provide evidence that the upregulation of HtrA1 in chondrocytes affected by OA substantiates its involvement in the development of the disease.

Multiple studies have provided evidence to support the notion that the infiltration of immune cells plays a substantial role in both the occurrence and advancement of OA [42]. The assessment and characterization of the variety of immune cells that infiltrate the affected area are of utmost importance in understanding the underlying molecular mechanisms of OA and developing novel targets for immunotherapy. Nevertheless, the precise functions and contributions of different immune cells within the microenvironment linked to OA remain unclear. As a result, we conducted an immune cell infiltration analysis on the samples. The findings indicate that there is dysregulation in the infiltration levels of various immune cell types, including activated dendritic cells, immature dendritic cells, Th1 cells, Th2 cells, Th17 cells, Tfh cells, γδT cells, Treg cells, central memory CD8 T cells, activated B cells, memory B cells, eosinophils, CD56dim natural killer cells, and MDSCs, within OA cartilage. Dendritic cells (DCs) consist predominantly of both immature and mature cells, functioning as antigen-presenting cells [43, 44]. According to the literature, it has been observed that immature dendritic cells have the ability to secrete both inflammatory cytokines and immunosuppressive cytokines [45]. Conversely, mature dendritic cells have been found to secrete inflammatory cytokines as well as immunostimulatory cytokines [45]. Cytokines, including TNF-α, IL-1β, IFN, and IL-6, which are secreted by DCs, play a substantial role in the development of OA [46]. Furthermore, several studies have provided evidence that DCs can enhance the levels of MMP-1 in synovial fluid [47]. B lymphocytes originate from pluripotent stem cells found in the bone marrow and are closely associated with OA. Patients diagnosed with OA commonly exhibit a notable presence of B lymphocytes within the synovium, and there is often a positive association between the extent of this infiltration and the level of synovial inflammation [48]. However, scholarly investigations have revealed that modifications occurring in the matrix at the chondro-osseous junction lead to a reduction in the generation of B lymphocytes and the manifestation of B lymphocyte cytokines [49]. Furthermore, the regulation of B lymphocyte levels is influenced by osteoclasts, and a reduction in B lymphocytes is observed as a symptom of osteosclerosis [50]. Herein, we observed that there was a decrease in the quantity of B lymphocytes within the cartilage affected by OA, potentially attributable to changes in the composition of the OA cartilage matrix. Given the intricate and multifaceted nature of OA, it is imperative to conduct additional research to explore the involvement of B lymphocytes in OA tissues. T cells, which constitute the predominant population of synovial infiltration in patients with OA, also exert a pivotal influence on the pathogenesis of this condition. The observation of CD4 + and CD8 + T cells within synovial aggregates is frequently reported [51]. In this study, the predominant immune cell populations found within the infiltrated cartilage of OA are regulatory T cells (Treg) and CD4 + T cell-derived subsets, including Th1, Th2, Th17, and helper follicular cells. T regulatory (Treg) cells are derived from undifferentiated T cells and possess the ability to modulate the release of anti-inflammatory cytokines, such as interleukin-10 (IL-10), as well as cytokine receptors [52]. The occurrence of inflammation is frequently accompanied by an elevation in the Th17/Treg ratio [53]. Nevertheless, several studies have indicated that although the secretion of IL-10 by Tregs in the peripheral blood of patients with OA is low, there is an increase in the quantity of Treg cells, potentially linked to a diminished Treg cell reaction [54]. The present study revealed an observed augmentation in the quantity of Treg within OA cartilage. However, additional investigation is required to elucidate the precise contribution of Treg in the pathogenesis of OA. Th cells and Tfh cells originate from CD4 + T cells, and various Th cell phenotypes are associated with different cytokines. The Th1 phenotype is associated with the synthesis of proinflammatory cytokines, which play a crucial role in the immune response against intracellular pathogens (as IFN-γ and IL-1β). The Th2 phenotype plays a crucial role in facilitating humoral immunity, regulating inflammatory processes, and contributing to the wound healing response. The primary cytokines associated with the Th2 subset are interleukin-4 (IL-4) and IL-13. The Th17 phenotype has the capability to generate IL-17, which in turn triggers an autoimmune response and stimulates the activation of neutrophils [55]. Follicular helper T cells have the capability to express a range of genes associated with inflammation and can additionally stimulate B cells to generate immunoglobulin [51]. An increasing body of scholarly literature indicates that TFH cells could potentially influence the severity of autoimmune diseases [including rheumatoid arthritis (RA]. However, the specific mechanism by which TFH cells operate in the context of OA remains uncertain. In addition to the aforementioned infiltration, there exists a presence of atypical T cells, specifically γδT cells. Several studies have provided evidence suggesting that γδT cells play a substantial role in the development and progression of RA [56]. While the precise mechanism by which non-traditional T cells contribute to OA remains unclear, it would be advantageous to explore the importance of these cells in the context of OA. Furthermore, it has been established that the dysregulation of CD56 natural killer cells, MDSCs, and eosinophils contributes to the onset and advancement of OA [56–58].

In addition, we investigated the potential association between genes characteristic of OA and dysregulated immune cells within the chondrocytes of patients diagnosed with OA. Tspan2 exhibits a correlation with γδT cells, Th2 cells, activated B cells, and memory B cells, whereas HtrA1 demonstrates an association with Th2 cells, Th17 cells, activated B cells, and memory B cells. This serves as the foundation for the involvement of OA-characteristic genes in the process of immune cell infiltration.

Nonetheless, our study does exhibit certain limitations. Firstly, given that OA exerts its influence across a spectrum of tissues, confining our assessment of immune cell infiltration solely to cartilage tissue possesses inherent constraints in portraying the complete engagement of immune cells in OA. Secondly, the correlation analysis of OA characteristic genes and infiltrating immune cells can only suggest a correlation, not reveal their causal relationship.

## Conclusions

Our investigation has revealed that osteoarthritis (OA) entails a multifaceted process influenced by numerous signaling pathways. The characteristic OA-related genes we identified, namely Herc5, Tspan2, and HtrA1, exhibit involvement in a diverse array of biological processes encompassing inflammatory responses, metabolism, and apoptosis. Furthermore, these genes are intricately linked to immune cell functions. This study not only serves as a wellspring of insight into unraveling the molecular underpinnings accountable for cartilage damage in OA but also furnishes a conceptual and practical foundation for the early identification and targeted therapeutic interventions of osteoarthritis.

### Supplementary Information

**Additional file 1.**

## Data Availability

All data and materials for this article are derived from the geo database and are genuinely available.The detailed website is as follows: https://www.ncbi.nlm.nih.gov/geo/query/acc.cgi?acc=GSE57218, https://www.ncbi.nlm.nih.gov/geo/query/acc.cgi?acc=GSE114007, https://www.ncbi.nlm.nih.gov/geo/query/acc.cgi?acc=GSE169077,
