# Supplementary material for: Identification of osteoarthritis-characteristic genes and immunological micro-environment features through bioinformatics and machine learning-based approaches
Source: BMC Med Genomics. 2023 Oct 7;16:236. doi: 10.1186/s12920-023-01672-y (PMC10559406; doi:10.1186/s12920-023-01672-y)
Supplement: Supplementary file 1 — Additional file 1. [file 12920_2023_1672_MOESM1_ESM.zip › Supplementary material/Explanation of supplementary files.docx]

- 1module_green
  - genes in module green.
- 2GO
  - GO enrichment analysis results.
- 2KEGG
  - KEGG enrichment analysis results.
- 3λ
  - λ-se and λ-min obtained from LASSO regression，and the number of genes screened for different λ values.
- 4diffgene
  - The list of DEGs and their logFCs.
- 5PCA.normalzie and 5PCA.preNorm
  - Principal component analysis before and after dataset merging.
