# Supplementary figures and images for: Identification of osteoarthritis-characteristic genes and immunological micro-environment features through bioinformatics and machine learning-based approaches
Source: BMC Med Genomics. 2023 Oct 7;16:236. doi: 10.1186/s12920-023-01672-y (PMC10559406; doi:10.1186/s12920-023-01672-y)

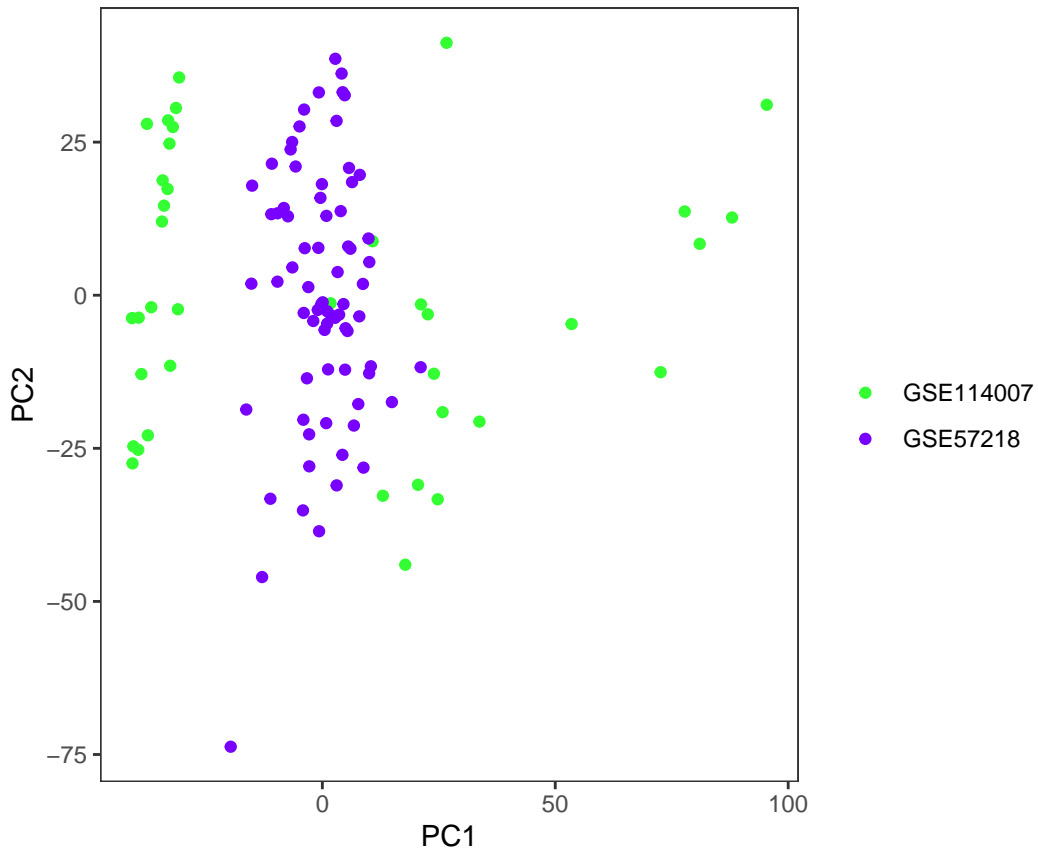

PCA diagram of the merged dataset of GSE114007 and GSE57218

Supplement: Supplementary file 1 — Additional file 1. [file 12920_2023_1672_MOESM1_ESM.zip › Supplementary material/5PCA.normalzie.pdf]

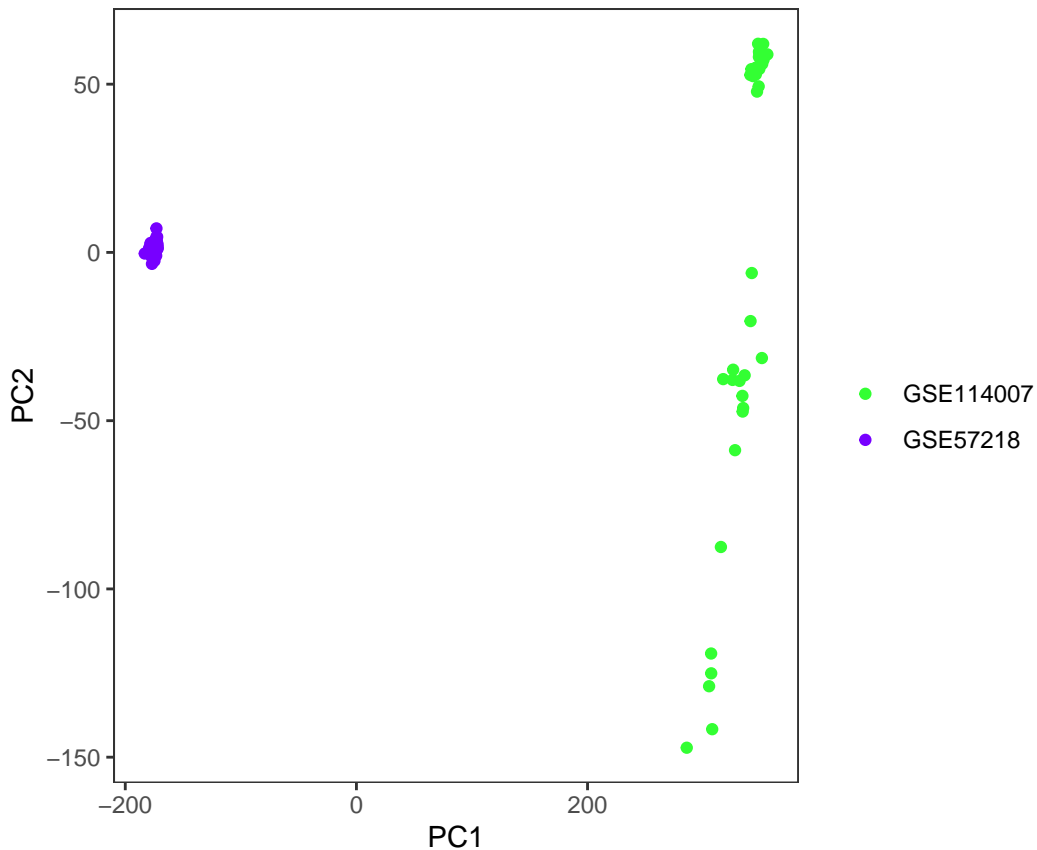

PCA image before merging datasets GSE114007 and GSE57218

Supplement: Supplementary file 1 — Additional file 1. [file 12920_2023_1672_MOESM1_ESM.zip › Supplementary material/5PCA.preNorm.pdf]
